# An in-depth interview study of women’s perspectives on the effects of contraceptive use on future fertility in Ethiopia and Kenya

**DOI:** 10.1016/j.ssmqr.2025.100624

**Published:** 2025-08-25

**Authors:** Kristen Kirksey, Hanna Feleke, Sarah Okumu, Hailemichael Bizuneh, Pauline Wekesa, Lauren Suchman, Beth Phillips, Zachary Kwena, Jenny Liu, Elizabeth Bukusi, Serah Gitome, Ewenat Gebrehanna, Kelsey Holt

**Affiliations:** a University of California, San Francisco, Institute for Health & Aging, 490 Illinois Street, 12th Floor, Box 0646, San Francisco, CA 94143; b Saint Paul’s Hospital Millennium Medical College, School of Public Health, 1271 Swaziland Street, Box 1271, Addis Ababa, Ethiopia; c Kenya Medical Research Institute, Center for Microbiology Research, Mbagathi Road, Box 54840-00200, Nairobi, Kenya

**Keywords:** Infertility, contraceptive side effects, contraceptive counseling, contraceptive agency, grounded theory

## Abstract

Infertility is a common perceived side effect of contraceptive use among women in sub-Saharan Africa, and fear of infertility can affect contraceptive choices. While no choice is inherently negative, it is critical that women have accurate information about side effects to ensure contraceptive decision-making agency. Despite the prevalence of infertility fears, little is known about women’s specific beliefs, where they originate, and how they inform contraceptive decisions. We sought to fill this gap in Kenya and Ethiopia through in-depth interviews with women aged 15–45 in Nairobi and Kisumu, Kenya, and Addis Ababa and North Shoa Zone of Amhara Region, Ethiopia (N=83). We utilized a modified grounded theory approach for data collection and analysis.

While not all participants believed in a link between contraception and infertility, those that did understood several mechanisms. Some, such as delayed return to fertility and aging out of childbearing years, were supported by clinical evidence. Others, such as accumulation of harmful substances in the body, damage to the reproductive system, and negative effects from early or prolonged use, were not supported by clinical evidence. Healthcare providers were a trusted source of information, disseminating information supported and unsupported by clinical evidence. Fear of infertility led women to prefer non-hormonal or short-term methods, avoid methods that cause amenorrhea, or delay use until after having children.

Results suggest the need to address side effect misinformation given by providers. Further, the specificity this study offers can inform strategies to improve contraceptive counseling and education campaigns in service of decision-making agency.

## 1. INTRODUCTION

Concerns about side effects play a key role in shaping women’s contraceptive decisions: in a global systematic review, “side effects and safety” was the most commonly reported issue raised in research on women’s values and preferences around contraceptive use (Yeh et al., 2022). While many side effect concerns, like menstrual bleeding changes, relate to effects supported by clinical evidence, others do not. Ensuring contraceptive programs and healthcare providers portray contraceptive side effects accurately, and that providers engage with women’s concerns respectfully and in a way that is aligned with their values and preferences, is critical to person-centered contraceptive care (Holt et al., 2020).

Infertility is a common perceived side effect of contraceptive use among women in sub-Saharan Africa, including in Ethiopia and Kenya, the sites for the current study. Nationally representative survey data demonstrate that 31% of women in Ethiopia and 21% in Kenya believe that contraceptive use could impact fertility (Bell et al., 2023; Zimmerman et al., 2024). Qualitative studies from Kenya, Ethiopia, and other African countries provide additional detail about this belief (Alvergne et al., 2017; Boivin et al., 2020; Polis et al., 2018; Sedlander et al., 2018, 2022). Most commonly, women believe that contraceptives cause infertility through an accumulation and blockage of blood; a buildup of chemicals or foreign materials; or structural damage to reproductive organs (Alvergne et al., 2017; Boivin et al., 2020; Sedlander et al., 2018). Bleeding changes, particularly amenorrhea, often invoke these fears (Polis et al., 2018). A woman’s reproductive history can impact her beliefs, with nulliparous women and younger women more frequently expressing a fear of infertility with contraceptive use (Boivin et al., 2020; Sedlander et al., 2018, 2022).

The fear of infertility with contraceptive use is persistent and deeply embedded socially and culturally. Some scholars link it to the historical emphasis on population control in global health, which led to coercive family planning programs and pervasive research abuses (Bau et al., 2024; Cottingham, 2015; Kaler, 1998; Steinke & Hövelmann, 2020). An ensuing legacy of distrust in many Global South countries has perpetuated the concern that infertility could be administered through health interventions (Kaler, 2009). Research from Kenya demonstrates that husbands’ or male partners’ fears of infertility, as well as social networks’ fears of infertility, influence women’s own fears and behaviors (Sedlander et al., 2021). Further, infertility can have significant impacts on women’s identities, social standing, and economic stability, and infertility treatments are inaccessible for many women, reinforcing the significance of this belief (Boivin et al., 2020; Greil et al., 2011; Meskelu & Berhane, 2018; Sedlander et al., 2018).

The fear of infertility is known to impact contraceptive preferences and use. A global systematic review demonstrated that impacts include low uptake and abstaining from contraceptives, switching to different or less effective methods, incorrect use of contraceptives, or using abortion to regulate fertility instead (Boivin et al., 2020; Woldetsadik et al., 2024). In Ethiopia, women report not intending to use long-acting and permanent methods due to fear of infertility (Gebremariam & Addissie, 2014). In Kenya, having a fear of infertility reduces odds of using contraceptives overall (Sedlander et al., 2021). While nonuse of contraception, use of less effective contraceptive methods, and abortion for fertility management are not inherently negative choices, it is essential that women have accurate information and support related to contraceptive side effects that align with their preferences to ensure these decisions are made with agency (Holt et al., 2024).

Prior qualitative studies offered limited depth into the belief that contraceptives cause infertility, including what contraceptive methods the belief applies to; what specific groups hold this belief; and the origins of beliefs (Stevens et al., 2023). We aimed to fill this gap through an in-depth interview study to explore women’s beliefs about contraception and future fertility in two countries in East Africa, Ethiopia and Kenya, chosen as examples of the socio-cultural diversity of the region. Specifically, we asked:

1. What are women’s perspectives on how contraception effects future fertility and what are the signs of this in Ethiopia and Kenya?
2. Where do women get information about the effect of contraception on fertility?
3. How do women’s perspectives on the effect of contraception on fertility impact their use of contraceptives?

We sought to identify commonalities and differences between Ethiopia and Kenya in addressing these questions. A deeper understanding of women’s beliefs about how contraceptive use may affect future fertility can provide context for values and preferences around contraceptive use and inform improvements to contraceptive programming and counseling to promote women’s contraceptive decision-making agency.

## 2. METHODS

We conducted a cross-sectional in-depth interview study in Kenya and Ethiopia. The aims of the overall study were to gain a deeper understanding of women’s experiences and preferences around contraceptive side effects, contraceptive counseling, and follow-up support for side effect management.

### 2.1 Study setting

The study took place in Nairobi and Kisumu, Kenya, and in Addis Ababa and North Shoa Zone of Amhara Region, Ethiopia. To include geographic, ethnic, and religious diversity, we selected two geographies in each country: the capital cities (Nairobi and Addis Ababa), with ethnically diverse urban populations, and one additional site - Kisumu, an urban area in western Kenya, and North Shoa Zone, a predominantly rural area in central Ethiopia. Unlike the urban sites, women in rural North Shoa Zone had lower education levels, more limited access to health facilities, and lived in a more religiously and culturally homogenous area (Bekele et al., 2022; EPHI and ICF, 2021).

### 2.2 Population and recruitment

Interviews with women (N=83) took place from April-May 2023. We took a purposive sampling approach to ensure diversity in the sample in terms of current and prior contraceptive use, method types, age, and marital status. We targeted approximately 40 women in each country based on prior research on women’s perspectives on contraception, as well as our aim to ensure adequate representation across the diverse categories of women in each country (Capurchande et al., 2016; Harrington et al., 2021). Eligibility criteria included being sexually active and a woman between ages 15–45. We interviewed both users and non-users of hormonal contraceptives. Recruitment was carried out in public healthcare clinics and through referrals from community health workers. In clinics, women were approached by the data collectors and enrolled in the study as they visited family planning service. Community health workers shared contact information of potential participants with research staff, who contacted them via phone to assess eligibility.

### 2.3 Instrument and data collection

The semi-structured interview guide contained questions aiming to gain a deeper understanding of women’s: 1) Experiences with and preferences regarding contraceptive side effects and 2) Preferences for enhanced support for side effect counseling during method selection and for supportive follow-up. For this analysis, we focused on data related to women’s perspectives on the relationship between contraception and fertility. Data came from women’s responses to several broad questions about experiences with and preferences related to side effects, as well as their responses to a probe on their thoughts about the relationship between contraception and infertility.

Interviews were conducted at locations and times chosen by the participants, with most taking place in private spaces in health facilities. They typically lasted 1–2 hours and were recorded using digital audio recorders. Interviews were conducted by trained research assistants who were all women and fluent in English and local languages (Kiswahili and Dholuo in Kenya and Amharic in Ethiopia). We remunerated participants ~10 United States dollars for their time (1000 Kenyan shillings or 500 Ethiopian birr).

The audio-recordings of the in-depth interviews were uploaded to an encrypted server. They were then transcribed and translated to English (where necessary) by either a team of experienced independent transcriptionists or the research assistants who conducted the interviews.

Research assistants obtained informed consent from all participants prior to their enrollment in the study. In Kenya, the study team obtained ethical approvals from the Maseno University Scientific Ethics Review Committee (MUSERC) (MUSERC/1171/22) and the National Commission of Science and Technology Innovation (NACOSTI) (NACOSTI/P/24/33360). In Ethiopia, we obtained approval from the Institutional Review Board (IRB) of Saint Paul’s Hospital Millennium Medical College (SPHMMC) (PM23/487). In the United States, we received ethical approval from the IRB of the University of California, San Francisco (22–37961).

### 2.4 Analysis

We took a modified grounded theory approach to data collection and analysis (Charmaz, 2014). Concurrent with data collection, we conducted ongoing rapid analysis of the data, using a previously established approach to coordinating a modified grounded theory study across multiple settings (Suchman et al., 2023). Research assistants completed data collection memos after each interview, which included participant’s experiences and preferences with side effects, side effects management, concerns around bleeding changes and infertility, any notable emerging themes, and recommendations for changes to the interview guides. We held weekly meetings in each country, where team members discussed emerging themes arising from memos and their review of select full transcripts. We held biweekly meetings with the full three country team, where we discussed similarities and differences among findings from each country and agreed on where to add probes in subsequent data collection.

This modified grounded theory approach allowed for the adjustment of sampling targets for remaining interviews to ensure we reached thematic saturation and the opportunity to iteratively develop interview guides, as allowable by IRBs.

We began coding with a provisional codebook, with codes informed by the rapid analysis. Per the grounded theory approach, a team of experienced coders from Kenya, Ethiopia, and the United States conducted initial coding of interview transcripts. Prior to the start of coding, the coding team completed an intercoder reliability exercise to ensure that they understood and consistently applied the codes. Through weekly analysis meetings, we discussed emerging findings and refined codes to iteratively develop the codebook, resulting in 27 parent codes.

At the end of initial coding, we had one parent code for infertility. A smaller team of coders began axial coding with three sensitizing concepts, informed by the research questions: beliefs around infertility and contraceptive use, sources of beliefs, and impacts on contraceptive use. These sensitizing concepts offered an analytic frame for the remainder of the analysis (Bowen, 2006). Themes were developed and refined throughout axial coding.

We opted to omit selective coding from our modified grounded theory approach. This decision was made to allow for the exploration of a broader range of themes, rather than focusing on the development of a single core theory. By doing so, we aimed to provide a more descriptive analysis that captures the diversity and complexity of the data, aligned with our research aims of detailing women’s beliefs related to contraception and infertility.

## 3. RESULTS

### 3.1 Participant characteristics

Table 1 presents the descriptive characteristics of the study sample, providing an overview of key demographic variables, as well as contraceptive use. Most participants were aged 20–45 and already had children. In Ethiopia, most participants were married (88%), while in Kenya nearly half were married and half unmarried. Fifty-eight percent of study participants were contraceptive users, while 42% were non-users. Users were defined as women who have used or are currently using a contraceptive method. In Kenya, this included hormonal and non-hormonal (e.g. withdrawal, condoms, fertility awareness method) contraceptive methods, while in Ethiopia, this included only hormonal contraceptive methods and the copper IUD. In Kenya all participants were recruited from urban study sites, while in Ethiopia half were recruited from urban sites (Addis Ababa) and half were recruited from rural sites (North Shoa zone).

### 3.2 Mechanisms underlying the fear of infertility include some supported by clinical evidence and some not.

**Figure 1.**
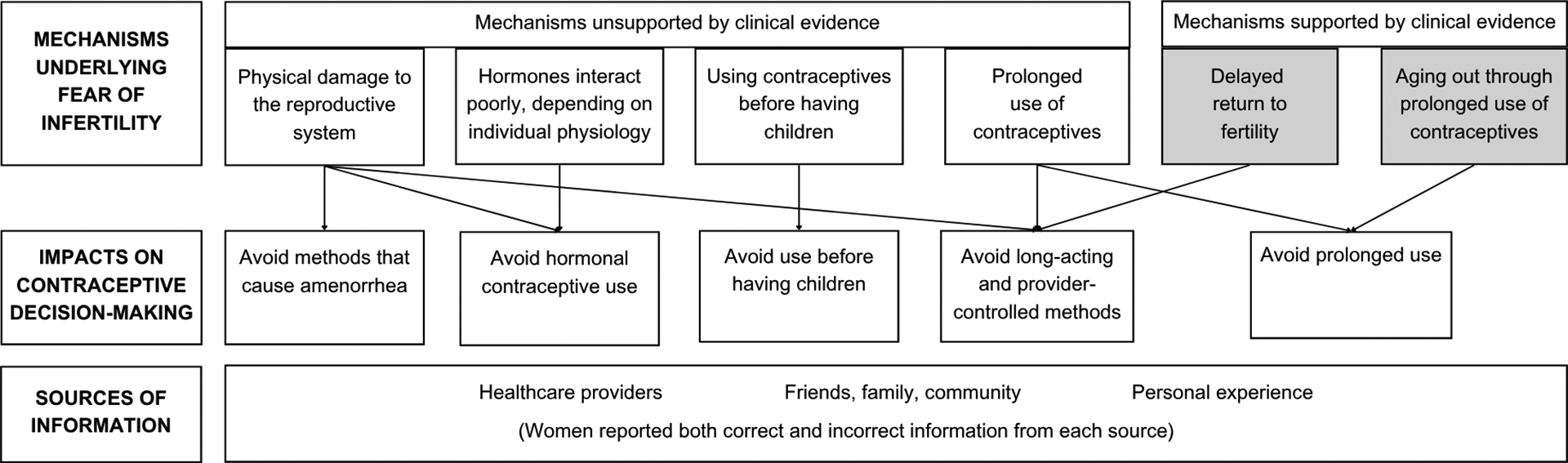
The belief that contraceptives cause infertility: Underlying mechanisms, impacts on use, and sources of information.

The data revealed diverse beliefs around contraceptive use and future fertility. While some individuals expressed confidence in the safety of contraceptives, many others feared they could cause infertility. Women who had a fear of infertility understood there to be several underlying mechanisms. Some of these mechanisms—including contraceptives causing physical damage to the reproductive system, using contraceptives before having children, and prolonged use of contraceptives—were not supported by clinical evidence. Others, including delayed return to fertility and aging out of childbearing years, were supported by clinical evidence. Figure 1 demonstrates mechanisms underlying the fear of infertility; how a belief in a specific mechanism impacts contraceptive use; and sources of information on contraceptive use and future fertility.

#### 3.2.1 Mechanisms not supported by clinical evidence include contraceptives causing physical damage to the reproductive system, using contraceptives before having children, and prolonged use of contraceptives.

Many women believed that contraceptives cause physical damage to the reproductive system, leading to infertility. Some women, particularly those in Kenya, believed that this damage happens through the accumulation of contraceptives or their byproducts. This belief was usually associated with the use of contraceptive pills, as they are taken daily, and thus, more likely to accumulate. A participant describes this belief, attributing the physical damage to the reproductive system to a buildup of metals:

They said if you take a lot of [those] drugs, it doesn’t really dissolve in the stomach. I don’t know where it goes. But then the metals that are inside there, they get deposited in your uterus and now you will have problems in having kids.

(Kenya-Kisumu, user, 20–45, unmarried)

Another participant describes how this belief impacts her decision to use a non-hormonal contraceptive method, common among participants who hold this belief:

Someone told me - there was just a discussion among girls - that those pills do accumulate in the stomach and can lead to infertility. Or when you want a child, it can take you 20 years. So, I stopped and was just using condoms.

(Kenya-Kisumu, user, 20–45, unmarried)

Other women in both countries believed that the physical damage that leads to infertility occurs through blockages in the reproductive system. Women often mentioned “blocked tubes” and “clots.” This belief was most commonly associated with contraceptive methods that cause amenorrhea, like implants and injectables.

Women in both countries also believed that contraceptives cause damage to ovaries, uterus, or eggs, causing infertility or miscarriages. Some believe this occurs through direct physical damage, and others believe that hormones or foreign chemicals in the body lead to the damage. This belief was associated with virtually all hormonal methods and led many participants to avoid contraceptives altogether. This participant describes her belief, an important factor in her decision not to use contraceptives:

Anytime you are menstruating and never giving birth, the ovum is being destroyed. So it causes fertility issues and you cannot give birth.

(Kenya-Kisumu, non-user, 15–19, married)

Participants in both countries raised two concerns of particular relevance for younger women, who may want to delay fertility for longer periods: using contraceptives before having children and prolonged use of contraceptives. These concerns highlight that it is not only the contraceptives themselves that cause concern for women, but also how they are used.

The belief that using contraceptives before having children could cause infertility was notably reinforced by healthcare providers. This concern was most frequently linked to the injectable, leading younger users to avoid it before having children and prompting others to advise young women to do the same. A participant elaborates on the mechanism:

As I heard from different people taking contraceptive will lead to infertility especially if you use contraceptive before giving birth to your first child. If it is before giving birth, the hormone will increase in your blood and I agree with this social perspective

(Ethiopia-Addis Ababa, non-user, 20–45, married).

The belief that prolonged use of contraceptives could lead to infertility was associated with all hormonal methods, but there was particular concern around long-acting reversible contraceptive methods, including IUDs, implants, and injectables. This belief led women to avoid prolonged use of contraceptives:

Yes, I have my worries about it. In some families, the women use contraceptives repeatedly and for a long time. I have heard that it could cause infertility. You need to use it for a brief time

(Ethiopia-North Shoa Zone, non-user, 15–19, married)

Other women concerned about prolonged use avoided provider-controlled methods, as they preferred to maintain control over decision-making.

Some participants who made an association between contraceptive use and fertility believed that contraceptives’ impact on fertility depends on the individual. This belief was associated with all hormonal contraceptive methods and led many women to avoid hormonal contraceptive use altogether. Some mention an individuals’ hormones or genetic makeup as determining factors:

You will find that there are those who become infertile completely as a result of using contraceptives. There are those however who become pregnant immediately after discontinuing the use of family planning. I feel that it depends [on] an individual’s hormones

(Kenya-Nairobi, non-user, 15–19, married)

Others believe more generally that it just depends: “I believe it depends on the person. What works on others may not work on me” (Ethiopia-Addis Ababa, non-user, 20–45, Married). Participants source this information from their own or others’ experiences:

[Contraceptives] might spoil your womb, might spoil your eggs. Two of my sisters have used it for a long time and they both have children. There is [another person] who used and now it’s almost five years and she has not been able to get a child…It depends on your eggs and how your body would stand depo.

(Kenya-Nairobi, non-user, 15–19, married)

#### 3.2.2 Not all fears related to future fertility are unfounded, including delayed return to fertility and aging out of childbearing years.

While many of the mechanisms that underlie a fear of infertility were not supported by clinical evidence, others were in fact grounded in clinical evidence. Women in both countries correctly pointed out that some contraceptives can cause a delayed return to fertility. Women expressed concerns about delays in conception ranging from three months to several years, based on their own experiences or those of others. This concern was most associated with injectables and implants and led women to avoid long term and provider-controlled methods. One woman described what she had heard in her community:

They say that the three-month injection will delay pregnancy after you use it for a long time, and you will not get pregnant when you want to. If it was used up to three times, it could delay by three months. Some people have delays for an entire year.

(Ethiopia-Addis Ababa, user, [age missing], married)

Women in both countries believed that prolonged use of contraceptives impacted fertility not through a direct physiological mechanism, but instead indirectly by aging out. Women expressed that your “age will pass to give birth” or “you might grow old and not be able to bear a child” after prolonged use. A woman in Kenya addressed this belief: “Yes, a lot of women say that contraceptive makes women to fail to give birth, but that is not the case. It is just that maybe they forget there is menopause” (Kenya-Kisumu, user, [age missing], married). One woman highlighted the tradeoff between economic stability, an oft-cited benefit of contraceptive use, and the potential loss of fertility through aging:

Yes, after using [contraceptives] for a while, your life becomes better, and you want to have a child, but you are unable to do so because you will be 50 or 40 years old. So it’s about aging, not the contraceptive.

(Ethiopia-North Shoa Zone, user, [age missing], married)

### 3.3 Some women did not view contraceptive use as linked to future fertility.

While the belief that contraceptive use causes infertility is common in Kenya and Ethiopia, we also found that some women did not hold this belief. This was driven by the feeling of limited control over fertility, trust in authoritative information, or already having children.

#### 3.3.1 Women believe fertility is beyond their control, reducing fear.

Many women, particularly those in Ethiopia, invoked “God’s will” in discussions about fertility. They believed that fertility was up to God and beyond their control, which mitigated concerns about contraceptive use. Participants had perceived a range of experiences with fertility after contraceptive use among women in their communities, with some having an immediate return to fertility, some experiencing a delayed return, and others to having permanent infertility. Therefore, they did not attribute fertility to an individual’s contraceptive use, but instead to God. One woman emphasizes: “I said that God could make you infertile, but not by the family planning method.” (Ethiopia-North Shoa Zone, non-user, 20–45, married).

#### 3.3.2 Women’s trust in research and government health authorities reduces concerns about infertility.

Many participants, particularly those in rural North Shoa Zone, Ethiopia, expressed confidence in the safety of contraceptives, underpinned by their trust in research and government health authorities. They did not believe that these authorities aimed to intentionally cause infertility and felt that sufficient research has been carried out on contraceptives to ensure their safety. This trust in authority, including healthcare providers, is especially prevalent among rural participants, reflecting both the lack of alternative sources of healthcare information and family planning care, and broader cultural norms supporting trust in public institutions (Asfaw, 2019):

[I never thought that the contraception method can make me infertile] Because there were studies and experiments before they made women use contraception. I don’t think anything that comes from doctors is problematic. I trust [the doctors].

(Ethiopia-North Shoa Zone, user, [age missing], married)

#### 3.3.3 Multiparity reduces women’s concerns about infertility.

Women in both countries expressed minimal concern about fertility because they already had children, which reduced the significance of future fertility. This does not imply that they lacked an underlying belief that contraceptives could cause infertility. Rather, they were not fearful of it, which made them more comfortable with contraceptive use.

After I had my daughter, I thought even if I become infertile now, I already have a daughter. I think that also made me very comfortable. And I think that is also one of the reasons that pushed me to now get an implant because I was like, if these side effects include being infertile…at least I already have a daughter.

(Kenya-Nairobi, user, 20–45, unmarried)

### 3.4 Information about future fertility, both supported and unsupported by clinical evidence, moves through multiple channels.

Information both supported and unsupported by clinical evidence moves through multiple channels, including healthcare providers, community members, and personal experiences. As each of these was a trusted source, the information they provided had a significant influence on women’s contraceptive preferences.

#### 3.4.1 Health care providers are a trusted source of information, and often spread information not supported by clinical evidence.

Healthcare providers in both countries were a trusted source of information on contraceptive use and fertility. Many women referenced accurate information from providers, reassuring them that they should not fear contraceptives:

When [healthcare providers] were telling us about contraceptives, questions about infertility were raised. They explained that [contraceptives are not related with infertility], and they said don’t worry if we want to have children; you will remove it and you can have children. This is for you to make gaps. Because they told us about this, we have no fear that it will cause infertility. It’s removed from our community consciousness.

(Ethiopia-North Shoa Zone, non-user, [age missing], married)

While many healthcare providers disseminated information supported by clinical evidence, and addressed incorrect information present in communities, others spread inaccurate information about contraceptive use and fertility, primarily in Kenya. Commonly, healthcare providers told women that using contraceptives before having children, particularly injectables, would cause infertility later in life, a common concern for younger users.

While in school there is a doctor who came and explained to us that if you are a student, you are not supposed to use the injection before giving birth. It will interfere with the eggs in your stomach, and it will make you not have babies. He mentioned its okay to use it once you have given birth at least once.

(Kenya-Kisumu, User, 15–19)

#### 3.4.2 Friends, relatives, wider community serve as a trusted source of information.

Friends, relatives, and the wider community served as key sources of information about contraceptive use and fertility in both countries. Many women reported hearing about an association between contraceptive use and future fertility through one-on-one conversations with close female friends and relatives. Others report learning about it during informal “discussion among girls” or “chitchat.” Other times, women gleaned information by observing and interpreting the experiences of those in their communities, both close female friends and relatives, as well as looser connections like neighbors and acquaintances. One woman described what she observed in her community and how it impacted her beliefs:

I remember a woman who gave birth after 17 years of having her first child. If the family planning method was going to make her infertile, she would have been infertile. So, the concept that family planning makes you infertile is not true.

(Ethiopia-North Shoa Zone, non-user, 20–45, married)

#### 3.4.3 Personal reproductive history is a source of information and preferences.

In addition to family, friends, and community, personal reproductive history was also an important source of information on contraceptive use and fertility and impacted participants’ preferences and use. Personal experience of giving birth after using contraceptives, even with prolonged use, gave women confidence that contraceptive use does not affect fertility. These personal experiences often outweighed information heard in their communities and reduced fears around contraceptive use. Alternatively, personal experience with delayed return to fertility after contraceptive use reinforced concerns about infertility.

### 3.5 The fear of infertility influences contraceptive decision-making.

A fear of infertility had notable impacts on women’s preferences around contraceptive use (Figure 1). Regardless of whether they are supported by clinical evidence, women’s beliefs and the mechanisms underlying them, have overlapping impacts on contraceptive use. This includes which methods clients choose, avoiding hormonal methods, long-acting or provider-controlled methods, and methods that cause amenorrhea. It also impacts how women used contraceptives, avoiding prolonged use or use before having children.

#### 3.5.1 Fear of infertility impacts which methods women choose.

Virtually all hormonal methods were raised as being associated with infertility through varying mechanisms. Participants who believed that hormonal contraceptives cause physical damage to the reproductive system through the accumulation of hormones or harmful chemicals avoided hormonal contraceptive use altogether. These women instead opted for non-hormonal methods like condoms or the copper IUD.

With me, I always fear using hormonal or any other type apart from condom because I always feel maybe I can fail to get babies in future as a side effect of the contraceptives. [Hormones] are new things in my body so I always feel like they can harm my body or change my cycle.

(Kenya-Kisumu, non-user, 20–45, unmarried)

Participants who believed contraceptives’ impacts on fertility depend on the individual also avoided hormonal contraceptive use altogether.

Other women believed that contraceptives cause physical damage to the reproductive system through blocked blood. Women who believed this mechanism avoided methods that cause amenorrhea, including implants and injectables. For many of these women, regular menstruation was perceived as a sign of fertility, so amenorrhea led to concerns around infertility.

If the [menstruation] stops while the needle is in, I will take it out because I want to give birth. I would rather take it out and change it to something else. If [menstruation] disappears altogether, it will lead to infertility.

(Ethiopia-North Shoa Zone, user, 20–45, married).

Women who believed physical damage to the reproductive system and prolonged use of contraceptives as mechanisms causing infertility avoided long-acting and provider-controlled methods. Short-term methods allow them to discontinue or switch methods without needing to consult healthcare professionals if they experience concerning side effects, thus maintaining control over their contraceptive decision-making.

I heard people say that the 3- and 5-year implant stops your period and shuts your womb…So, the thought of it scared me, and that’s why I don’t prefer such long-acting contraceptives. I only wanted the 3-months because it’s easier to discontinue if I find a problem with it.

(Ethiopia-North Shoa Zone, user, [age missing], married)

Women who had experienced or observed delayed return to fertility with contraceptive use also avoided long-acting and provider-controlled methods, specifically injectables and implants.

#### 3.5.2 Fear of infertility impacts how women use their chosen contraceptives.

Fear of infertility not only impacted which methods women chose, but also how they used those methods. Women who believed that prolonged use of contraceptives impacts fertility, either directly through reproductive harm, or indirectly through aging, avoided prolonged use. One woman described using contraceptives short-term with the intention of birth spacing:

I never thought I would use [contraceptives] for a long time. I only need it for a few months until my newborn gets strong. What I heard is that women who use pills or injectable for 3 or 4 years will face [fertility] problems.

(Ethiopia-Addis Ababa, user, 20–45, married)

Other women, hearing from healthcare providers or their communities, that using contraceptives before having their first child impacts fertility, preferred not to use contraceptives before having children and advised other women on the same.

## 4. DISCUSSION

This paper details women’s perspectives on contraceptive use and future fertility, describes sources of information, and details the impacts of this fear on contraceptive preferences among a diverse sample in Kenya and Ethiopia. Aligned with existing literature, the belief in infertility as a side effect of contraceptive use was common, though not universal, in our sample (Bell et al., 2023; Zimmerman et al., 2024). The findings demonstrate, however, that women’s fear of infertility is not a blanket fear. It is made up of a patchwork of beliefs: contraceptives cause infertility for some women, using some methods, some of the time. They can cause primary and secondary infertility, as well as delayed return to fertility and permanent infertility. Some aspects of this belief are supported by clinical evidence, and some are not. A deeper understanding of how women specify this belief – and the fact that some portion of the concern is justified — is a key contribution of this paper. A fear of infertility impacts if women use contraceptives, which methods they choose, and how they use their chosen methods. Though hormonal contraceptives remain the most used methods among contraceptive users, with 94% of women in Ethiopia and 76% of women in Kenya relying on them (ICF, 2019, 2022), our findings demonstrate that the fear of infertility remains a salient concern for women in both countries.

Women who believed contraceptive use was associated with future infertility understand there to be several underlying mechanisms. Some mechanisms, such as the accumulation of harmful substances in the body, physical damage to the reproductive system, and negative effects from early or prolonged use, are not supported by clinical evidence, aligning with prior findings (Bell et al., 2023; Boivin et al., 2020; Sedlander et al., 2018, 2022). A unique finding from this study was the belief that hormones interact with an individual’s particular physiological constitution, causing harm for some women but not others.

On the other hand, some women’s fear of infertility stemmed from accurate concerns about the indirect link between using contraceptives for a prolonged period and aging out of one’s childbearing years. Further, women in our study highlighted that contraceptive use can cause a delayed return to fertility, in line with research demonstrating that injectables and implants can delay the return to fertility for over a year after discontinuing use (Gemmill et al., 2023). While a relatively short delay tends to be viewed by clinicians and researchers as insignificant over the course of one’s life, it is important to remember that for some women, the difference between “delayed return to fertility” and “infertility” may be insignificant, and that delayed return to fertility can have interpersonal and material impacts, particularly for women later in their reproductive years (Boivin et al., 2020; Stevens et al., 2023). Women who did not desire more children were less likely to express fears about infertility, suggesting that fear of infertility is particularly relevant for the large proportion of women who do desire children in the future—64% in Ethiopia and 57% in Kenya (ICF, 2016, 2022).

Although healthcare providers were trusted sources of information in our study, women reported sometimes receiving information not supported by clinical evidence, particularly in Kenya. Existing research often places the responsibility for “misinformation” on women and emphasizes addressing it at the individual level. However, this paper aligns with other studies showing that misinformation is more systemically distributed through providers (Boivin et al., 2020). Thus, strategies to address it should be multi-level and prioritize addressing systemic issues that lead to spread of incorrect information via healthcare providers. As these findings reflect women’s experiences and interpretations of counseling, rather than an objective assessment of the accuracy of information conveyed during counseling, future research could use observational approaches to better understand how messages about contraceptive use and future fertility are communicated, and which types of providers may be more likely to disseminate incorrect information. Interventions should recognize that providers themselves are embedded in community and bring their own interpretations of their own and others’ experiences to counseling. It is also important to consider the paternalism and power imbalances in the patient-provider interaction that prevent women from asking questions and pushing back on inaccurate information from providers (Holt et al., 2020).

In addition to addressing active spread of misinformation, improvements to contraceptive counseling approaches are needed to better integrate an acknowledgement of women’s valid concerns about infertility when they have them. Because many of women’s beliefs around contraceptive use and fertility are not supported by clinical evidence, dominant discourse in the contraception field frames a fear of infertility as myth, rumor, or misinformation, and focuses on correcting women (Ankomah et al., 2011; Diamond-Smith et al., 2012; Jonas et al., 2022; Sedlander et al., 2022; Stevens et al., 2023). However, there is inadequate research on women’s beliefs around contraceptive use and future fertility to broadly label them as myth or misinformation, and interventions aiming to change these beliefs have been largely ineffective (Stevens et al., 2023). Because women have various understandings of the mechanisms that underlie contraceptive use and fertility, they require not just an overarching statement that contraceptives do not cause infertility, but rather a more nuanced explanation of the mechanisms underlying contraceptive use. This should be shared in a manner that resonates with them and contextualizes their observations and experiences. Taking women’s beliefs into account, rather than aiming to change them may be a more effective approach to support women in meeting their contraceptive needs (Stevens et al., 2023).

### 4.1 Recommended counseling and research approaches to address beliefs around contraceptive use and future fertility

Some specific examples of counseling approaches informed by findings from this study include:

- Recognizing that women have experiences and valid concerns around delayed return to fertility, guidance on return to fertility for each method should be a key component of counseling.
- Recognizing the pervasiveness of the belief that prolonged use or use of contraceptives before having children can lead to infertility, providers should emphasize that this is untrue, particularly for younger women.
- Recognizing that women may believe contraceptives can cause infertility for some women and not others, counseling should clearly reassure them that contraceptives do not cause infertility for any woman, regardless of her physiological constitution.
- Recognizing that fear is sometimes driven by concerns about hormones, providers should be sure to include non-hormonal methods in counseling. This can ensure that women likely to choose methods such as withdrawal or fertility awareness are well equipped to use them rather than left without information on how to use them effectively.
- Recognizing that many women fear an accumulation of foreign materials in the body as a cause of infertility, providers should offer clear counseling on the mechanisms of action of various contraceptive methods, validating their concerns that foreign substances enter into the body as part of many contraceptive methods, but clarifying that they do not harm reproductive organs.
- Recognizing that amenorrhea can drive fears of infertility, providers should be sure to counsel on methods that do not have this side effect profile and to be prepared to support switching when women using hormonal contraception return unsatisfied with their method.

Qualitative findings on infertility beliefs, such as those from this study, can also be used to improve quantitative measures with items that probe on specific beliefs. Such measures would be useful in programmatic research aimed at developing and evaluating interventions and counseling approaches to address contraceptive side effect concerns.

### 4.2 Limitations

This study had some limitations. First, the phrasing of our interview question probing women directly about infertility was potentially leading (“Even though it is not medically accurate, some women are afraid to take contraceptives because they believe it will prevent them from having children in the future. Were you concerned about this?”) and may have introduced social desirability bias, prompting participants to align their responses with what they believed was expected by researchers. Further, by probing women directly about infertility concerns, we recognize there is a risk of unintentionally contributing to women’s concerns. Even when framed carefully, asking participants about a possible link between contraceptives and infertility could reinforce or create misconceptions about a possible association, especially in contexts where these fears are already prevalent. Second, the definition of infertility we used in the interview probe was general, allowing participants to interpret for themselves what preventing pregnancy “in the future” means. Future research could explore more in-depth women’s own definition of when delayed conception becomes an indication that a person is not able to have children. A third limitation was the lack of women recruited from rural areas in Kenya, which may have prevented us from capturing nuance in our findings relevant to this group.

## 5. CONCLUSION

This study highlighted that women’s beliefs about the impact of contraceptive use on future fertility stem from a wide range of understandings of underlying mechanisms, some grounded in accurate reproductive physiology and others not. Healthcare providers are an important source of information, disseminating both accurate and inaccurate information. An inaccurate understanding of the relationship between contraception and infertility can put women’s contraceptive decision-making agency at risk. Counseling and other educational interventions can draw from these findings, as well as broader research on women’s side effects beliefs, to ensure accurate information is conveyed and to develop tailored, person-centered approaches that are responsive to women’s perspectives and concerns.

## Figures and Tables

**Table 1 -:** Descriptive characteristics of sample (N=83)

	Ethiopia		Kenya	
	(n)	(%)	(n)	(%)
Contraceptive use				
Non-user	18	42	9	23
User	25	58	31	77
Age*				
15–19	6	19	13	36
20–45	26	81	23	64
Marital Status*				
Married	38	88	18	49
Unmarried	5	12	19	51
Children*				
No	5	12	6	18
Yes	37	88	28	82
Education*				
Less than HS	27	68	6	19
High School	7	17	14	44
Some college	0	0	2	6
College graduate	6	15	10	31
Study site				
Addis Ababa	22	51	-	-
North Showa	21	49	-	-
Nairobi	-	-	20	50
Kisumu	-	-	20	50
N	43		40	

* Responses were missing on age for n=15 participants, on marital status for n=3 participants, on children for n=6 participants, and on education for n=8 participants.
